# Microbes in Our Food, an Ongoing Problem with New Solutions

**DOI:** 10.3390/antibiotics9090584

**Published:** 2020-09-08

**Authors:** Birgit M. Prüβ

**Affiliations:** Department of Microbiological Sciences, North Dakota State University, Fargo, ND 58104, USA; Birgit.Pruess@ndsu.edu; Tel.: +1-701-231-7848

**Keywords:** food borne microbial disease, novel anti-microbials

## Abstract

Despite an increasing number of techniques that are designed to mitigate microbial contamination of food and the resulting food borne disease outbreaks, the United States and many other countries across the world continue to experience impressive numbers of such outbreaks. Microbial contamination can occur during activities that take place in the pre-harvest environment or in the processing facility post-harvest. Current treatments of food that are aimed at reducing bacterial numbers may be only partially effective because of the development of bacterial resistance, the formation of bacterial biofilms, and inactivation of the treatment compound by the food products themselves. This Special Issue will include basic research approaches that are aimed at enhancing our understanding of how contamination occurs throughout the food processing chain, as well as more immediate and applied approaches to the development and use of novel anti-microbials to combat microbes in food. Novel techniques that aim to evaluate the efficacy of novel anti-microbials are included. Overall, we present a broad spectrum of novel approaches to reduce microbial contamination on food at all stages of production.

## 1. Introduction

While the world may be focused on SARS CoV-2 and Covid-19 this year, infectious diseases by other organisms, including bacteria, fungi, or parasites continue to happen. In particular, a number of foodborne disease outbreaks occurred just this year in the United States and other countries. The Special Issue entitled “Development of Novel Anti-Microbials to Reduce Bacterial Contamination of Food” is dedicated to finding new solutions to an ongoing problem. With this Commentary, the Special Issue Editor outlines the ongoing problem and presents examples of current techniques to mitigate the problem, together with limitations of these interventions. Brief research summaries will be included for those Special Issue authors who have been in communication with the Special Issue Editor prior to submission of their article or who have submitted their article early.

## 2. The Problem

The Centers for Disease Control and Prevention in the United States (CDC; www.cdc.gov) list seven outbreaks of *Escherichia coli* (serotypes O103, O157:H7, O121, O26), 12 outbreaks of *Salmonella enterica* (serovars Enteriditis, Newport, Javiana, Dublin, Uganda, Concord, Carrau, Schwarzengrund, Infantis), four outbreaks of *Listeria monocytogenes*, and two outbreaks of *Cyclospora* between January of 2019 and August of 2020 (Table 1). Note that many of these outbreaks were not limited to or originated in the US. As one example, the *E. coli* outbreak associated with sunflower kits involved five US states and Canada; it included 10 reported cases, as well as 5 hospitalizations. As a second example, the papayas that caused the *Salmonella* outbreak had been imported from Mexico; the outbreak included 81 reported cases in 9 states and 27 hospitalizations.

There have been multiple outbreaks in many countries across the world. Among these, a multi-country outbreak of *E. coli* gained significant attention in Germany in 2011 [1]. Not only did this new *E. coli* O104:H4 cause 3,000 cases of diarrhea, accompanied by 830 cases of hemolytic uremic syndrome and 54 deaths, but it also spread across 12 European countries over the summer. Using newly developed tracing tools and network graphs, the origin of the strain could be traced back to fenugreek seeds that had been imported from Egypt in 2009 [2]. The virulence mechanism of the new O104:H4 serotype is still not fully understood and it is questioned whether the danger might still be “out there” [3]. Canada experienced a total of 18 outbreaks of *Salmonella* between 2015 and 2019 [4]. The incidence of listeriosis increased in South Africa, starting in June of 2017 to become the world’s largest outbreak of listeriosis by July of 2018 [5]. The WHO estimates that the global burden of foodborne diseases between 2007 and 2015 was considerable; especially in Africa and South-East Asia and for children under five years of age and persons of low income [6].

## 3. Current Solutions

**Figure 1:**
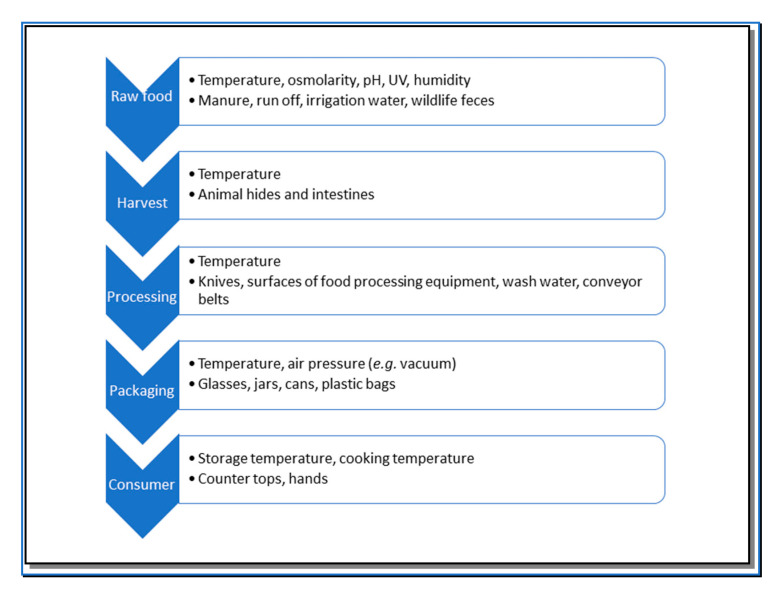
General schematic of a food processing chain. For each processing step, the first bullet lists environmental conditions that can impact presence of foodborne pathogens, and the second bullet lists sources of contamination.

A general schematic detailing the food processing chain is provided in Figure 1. A typical food processing chain consists of the raw animal or plant, slaughter (of the animal) or harvest (of the plant), processing in a facility, often packaging, and then distribution to the consumer. Figure 1 includes examples of environmental conditions that impact the presence of food borne pathogens, as well as common sources of microbial contamination. The raw food stage includes the pre-harvest environment, in which growing animals and plants are exposed to many environmental conditions and stressors, including but not limited to UV exposure, temperature, and humidity [7]. On plants, contamination by *E. coli*, *S. enterica*, and *L. monocytogenes* depends on irrigation water, manure, and wildlife feces [8]. To complicate matters, pre-harvest environmental conditions can increase resistance to post-harvest stresses, including those that bacteria will experience during processing and that are aimed at reducing their numbers (e.g., peroxyacetic acid [9]). At the harvest stage, transmission of pathogens can depend on the temperature within the facility, as well as humidity and aeration. When harvesting (slaughtering) animals, the hides and the intestines of cattle can be sources of contamination at harvest (slaughter) stage [10,11]. The next step, food processing within the facility involves many surfaces [12], including counter tops and conveyor belts. For plants, processing can involve consecutive rounds of wash water [13].

Interventions to inactivate microbes on food are diverse. At the food processing stage, they include chemical, physical, and biological treatments. For meat such as beef or chicken and certain vegetables including leafy greens, chemical treatment with organic acids is a common practice, often used in combination with other treatments. As one example, Carpenter and coworkers were able to reduce *E. coli* O157:H7 and *Salmonella* by 0.6 to 1 log/cm^2^ on beef and chicken meat surfaces by a combination of 2% levulinic acid with lactic acid or acetic acid [14]. On leafy greens, chlorine as well as range of other chemicals are used in the wash water to reduce cross-contamination [15]. Intriguingly, the efficacy of such treatments can be limited because of the inactivation of the free chlorine by fresh cut plant exudates, which facilitates cross-contamination during the wash cycles [16]. In addition, bacteria can form biofilms on many surfaces, including stainless steel, when treated with sub-lethal concentrations of the anti-microbial [17].

Among the biological treatments, bacteriophages have gained importance. As one example, a cocktail of six lytic bacteriophages was used to control *Salmonella* in pet food, so it can no longer be transmitted to humans [18]. As an example of biological treatment at raw food stage, bacteriophages have been administered to cattle orally and rectally to reduce shedding of *E. coli* O157:H7 [19]. In this case, the bacteria were unable to develop resistance to the phages.

At the consumer end, the CDC recommends to wash hands and surfaces often, keeping raw food products separate from ready to eat foods, cook to the correct temperature (e.g., 145 F for beef and pork, 165 F for poultry), and keeping food in the refrigerator at 40 F or below. While all these preventative techniques are undoubtedly helpful, outbreaks of food borne infectious disease continue to happen, and a need for novel and innovative approaches is evident.

## 4. Novel Approaches

The authors of this Special Issue address the above outlined need for the development of novel intervention techniques.

Dr. Valentina Trinetta from Kansas State University in the US studies a variety of foodborne pathogen bacteria, including *Salmonella typhimurium* monophasic, *Listeria monocytogenes*, and STEC *E. coli*. Her research focus on understanding pathogens’ ecology and identifying microbial entry routes into the food supply chain. One study described 16 different serotypes of *S. enterica* in feed mills, including *S. Agona*, *S. Mbandaka*, *S. Senfenberg*, and *S. Scharzengrund*, all of which are of a public health concern [20]. Dr. Trinetta’s work extends to the development and implementation of antimicrobial interventions to reduce and control foodborne pathogens on different foods. Her group’s contribution to this Special Issue will be a study on the effect of combinations of several antimicrobial strategies for the processing of strawberries.

Dr. Siyun Wang from The University of British Columbia in Canada pursues an interesting range of food safety related research. One of her studies described an oxidizing mixture of hypochlorite and hydrogen peroxide to reduce *E. coli* O157:H7, *L. monocytogenes*, and *S. enterica* from alfalfa and radish seed. The combination treatment reduced bacteria by 3 log, but was less effective when seeds had been stored for 4 weeks or longer [21]. Most recently, her group is conducting a series of research projects exploring the use of bacteriophages (i.e., viruses that infect bacteria) on inactivating foodborne pathogens on fresh produce and poultry products [22]. This group works on improving food processing and safe food handling for long term food security.

Dr. Christina Andrea Müller, together with Dr. Gabriele Berg and their research group from Graz University of Technology in Austria, specialize on the study of the plant microbiome. Recognizing the relevance of the plant microbiome and the metabolic pathways of the plant associated bacteria for the discovery of bioactive compounds, she described the potential of the moss microbiome in the discovery of such compounds (*Sphagnum* spp.) [23]. Another research article described the microbiome of edible plants of the genus *Brassica* and the isolation of bacterial strains of public health significance, in particular the prevention of cancer [24]. The new emphasis of her plant microbiome studies is the discovery of anti-microbials for applications in food production technologies.

Dr. Kalidas Shetty from the Department of Plant Sciences at North Dakota State University in the United States has over 35 years of teaching and research experiences. The goal of his research is the development of climate change-resilient food system innovations to combat global food and nutritional insecurity-linked public health challenges. He does this by studying the critical role of cellular and metabolic basis of redox-linked biology and dual functional roles of plant phenolics. Recent articles have highlighted the phenolic bioactive-linked anti-hyperglycemic and anti-bacterial properties in the herbs from the *Lamiaceae* family [25], as well as the antioxidant and anti-diabetic functionalities in Peruvian corn [26] and bioprocessed cashew apple juice [27]. The contribution to this Special Issue is from another research focus; metabolic-linked system innovation to enhance inducible phenolics with antimicrobial properties in food and medicinal plants for food safety and human health relevant applications. This manuscript is co-authored by Dr. Dipayan Sarkar and Ashish Christopher.

A research group including Bruno Kolb, Lorina Riesterer, Anna Maria Widenhorn, and Leona Bier from the Student Research Centre in Überlingen, Germany contributed an article to this Special Issue where monitoring the emission of hydrogen using a specific hydrogen sensor led to a recognition of contamination by *Borrelia* of cockerel and blood in the case of Lyme disease. This group of researchers has a background of similar research, utilizing headspace gas chromatography to monitor hydrogen and carbon dioxide for the purpose of determining efficacy of anti-microbials [28]. The same technique of headspace gas chromatography was used to metabolically profile *Borrelia* in blood [29]. Overall, the techniques by this research group aim at testing efficacy of anti-microbials.

## 5. Conclusions

This Special Issue covers an impressive array of novel techniques to reduce microbial contamination of food and food products. From anti-microbial treatments to phages, as well as genomics approaches to reducing bacterial contamination, we present a range of biologically derived interventions and include chemical techniques to determine the effectiveness of anti-microbials. These novel techniques are all fundamentally different from what is currently in use and once in application, should aid in the mitigation of food borne bacterial infections diseases.

## Figures and Tables

**Table 1:** Multi-state food borne infectious disease outbreaks in the US between January 2019 and July 2020.

Food/Product	Pathogen	Serotype	Month/Year
Clover sprouts	*E. coli*	O103	Apr 2020
Sunflower salad kits	*E. coli*	O157:H7	Jan 2020
Romaine lettuce	*E. coli*	O157:H7	Jan 2020
Northfork bison	*E. coli*	O103/O121	Sep 2019
Flour	*E. coli*	O26	Jul 2019
Ground beef	*E. coli*	103	Jun 2019
Romaine lettuce	*E. coli*	O157:H7	Jan 2019
Peaches	*Salmonella*	Enteriditis	Aug 2020
Onions	*Salmonella*	Newport	Aug 2020
Cut fruit	*Salmonella*	Javiana	Feb 2020
Ground beef	*Salmonella*	Dublin	Dec 2019
Papayas	*Salmonella*	Uganda	Sep 2019
Tahini	*Salmonella*	Concord	Jun 2019
Frozen tuna	*Salmonella*	Newport	May 2019
Pre-cut melon	*Salmonella*	Carrau	May 2019
Ground turkey	*Salmonella*	Schwarzengrund	May 2019
Tahini	*Salmonella*	Concord	Feb 2019
Chicken products	*Salmonella*	Infantis	Feb 2019
Ground beef	*Salmonella*	Newport	Mar 2019
Enoki mushrooms	*L. monocytogenes*		Jun 2020
Hard boiled eggs	*L. monocytogenes*		Mar 2020
Deli meat and cheese	*L. monocytogenes*		Sep 2019
Pork products	*L. monocytogenes*		Jan 2019
Bagged salad mix	*Cyclospora*		Jun 2020
Basil	*Cyclospora*		Sep 2019

Multi-state food borne disease outbreaks were taken from the CDC website on 23 August 2020 (https://www.cdc.gov/foodsafety/outbreaks/multistate-outbreaks/outbreaks-list.html).
